# Supplementary material for: Combination of a Novel Fusion Protein CD3εζ28 and Bispecific T Cell Engager Enhances the Persistance and Anti-Cancer Effects of T Cells
Source: Cancers (Basel). 2022 Oct 9;14(19):4947. doi: 10.3390/cancers14194947 (PMC9563022; doi:10.3390/cancers14194947)

cancers

MDPI

Supplementary Materials: Combination of a Novel Fusion Protein CD3εζ28 and Bispecific T Cell Engager Enhances the Persistance and Anti-Cancer Effects of T Cells

Feng Yu, Yang Gao, Yan Wu, Anran Dai, Xiaoyan Wang, Xiangzhi Zhang, Guodong Liu, Qinggang Xu and Dongfeng Chen

Materials and Methods

Generation of Hela-EGFRvIII BiTE/A549-EphA2 BiTE/K562-CD19

All the recombinant lentivirus were packaged and produced by transfecting the viral vectors into 293 T cells using Retrovirus or Lentivirus Packaging kit (VigenBio zhenjiang, Jiangsu, China) according to the manufacture instructions. Hela/A549 were mixed with lentivirus carrying EGFRvIII BiTE/EphA2 BiTE. For each tube, 50 μL concentrate of lentivirus were added into 2.5×10^5 cells in 100 μL DMEM medium. The mixtures were centrifuged at 400g/min for 50min. Then directly added 1mL DMEM medium containing 10% FBS into each tube, the cell suspension were seeded into 6 wells plate and cultured in 37°C 5% CO2 for further experiments. K562 were mixed with lentivirus carrying CD19. For each tube, 50 μL concentrate of lentivirus were added into 2.5×10^5 cells in 100 μL RPMI1640 medium. The mixtures were centrifuged at 400g/min for 50min. Then directly added 1mL RPMI1640 medium containing 10% FBS into each tube, the cell suspension were seeded into 6 wells plate and cultured in 37°C 5% CO2 for further experiments.

Cytotoxicity assay

A549-EphA2 BiTEs were co-cultured with T cells in 96-well plates for 24h. After removing the supernatant, the plates were gently washed twice with PBS. The viability of tumor cells were then determined using an MTS formazan viability assay (Promega, Fitchburg, WI, USA). Briefly, 5μL MTS solution and 95 μL medium were added into each well and incubated at 37°C for one hour. Optical density of each well was determined at 490 nm on a microplate reader (Molecular Devices VERSAmax).

Enzyme-linked immunosorbent assay (ELISA)

T cells were co-cultured with A549-EphA2 BiTEs at 24h. Then the supernatants were collected and analyzed by IL2/IFNγ(human) quantity ELISA kit (BD Biosciences) according to the manufacturer's instructions. Data were obtained by detecting the absorbance value of the sample at the indicated wavelength.

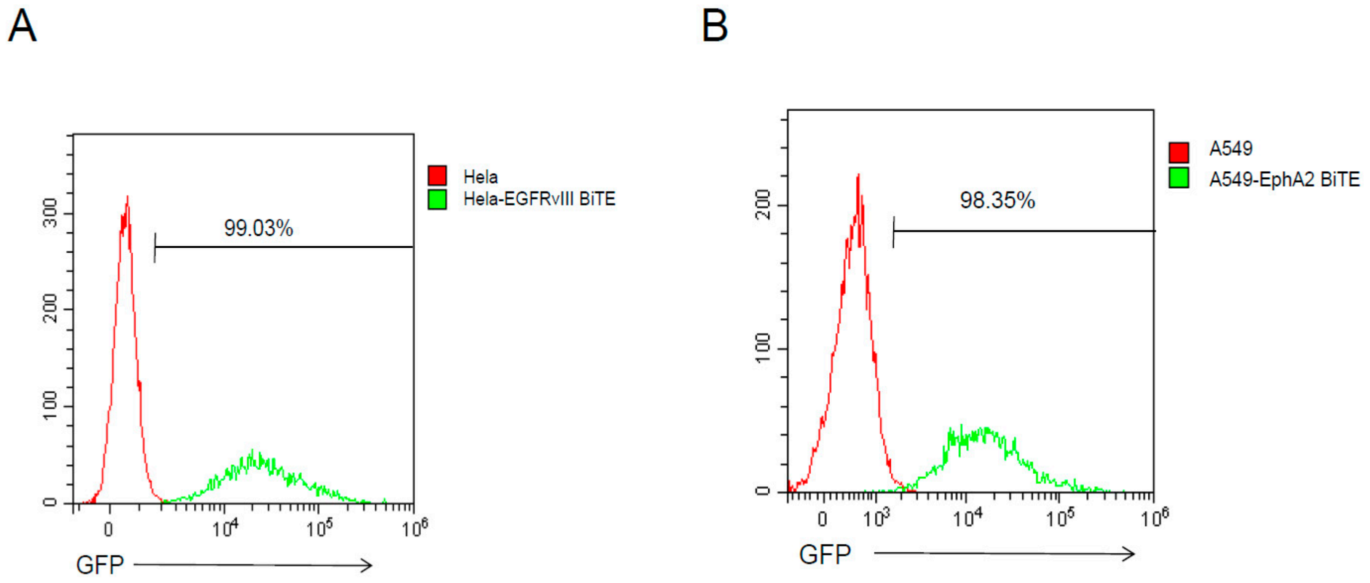

Figure S1. Generation of tumor stable cell lines. (a)Generation of Hela-EGFRvIII BiTE cells. Flow cytometry analysis showed the transduced rate of Lentivirus carrying EGFRvIII BiTE-GFP in Hela cells based on the expression of GFP. The wild type was a non-tranduced control. (b)Generation of A549-EphA2 BiTE cells. Flow cytometry analysis showed the transduced rate of Lentivirus carrying EphA2 BiTE-GFP in A549 cells based on the expression of GFP. The wild type was a non-tranduced control.

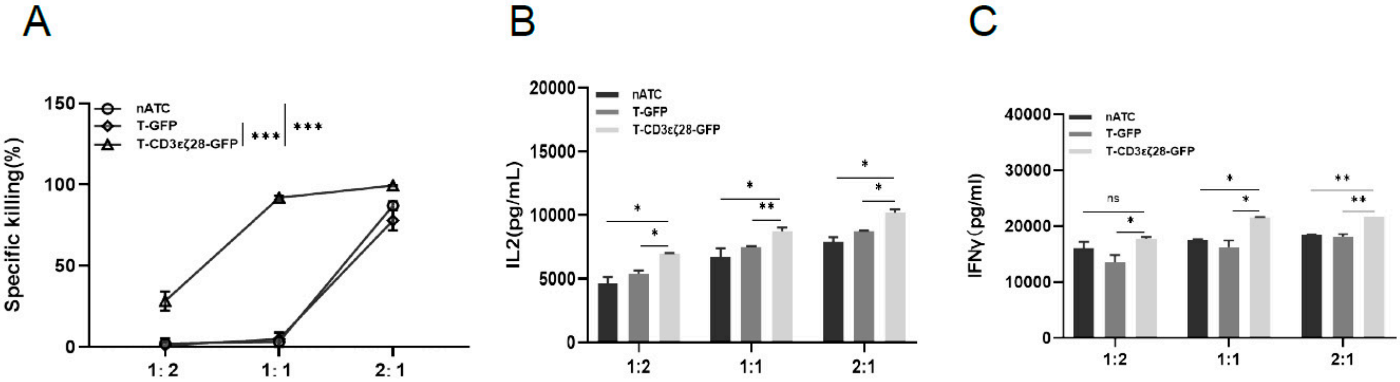

Figure S2. Comparision of the killing effects of different T cells on A549 cells expressing EphA2 BiTE. A549-EphA2 BiTE were co-cultured with nATC or T-GFP or T-CD3εζ28-GFP at different E:T ratios for 24 hours. The viability of cancer cells was determined by using MTS assays(a). (b) and (c) Histograms showed the production of cytokines in different T cells. A549-EphA2 BiTE cells were co-cultured with nATC or T-GFP or T-CD3εζ28-GFP at different E:T ratios for 24 hours, and then the supernants were collected. The concentration of IL2(b) and IFNγ(c) in supernants were detected by enzyme-linked immunosorbent assay (ELISA). Statistical differences were assessed by mutual comparison between each two groups using multiple comparisons t test. (*$p<0.05$, **$p<0.01$, ***$p<0.001$).

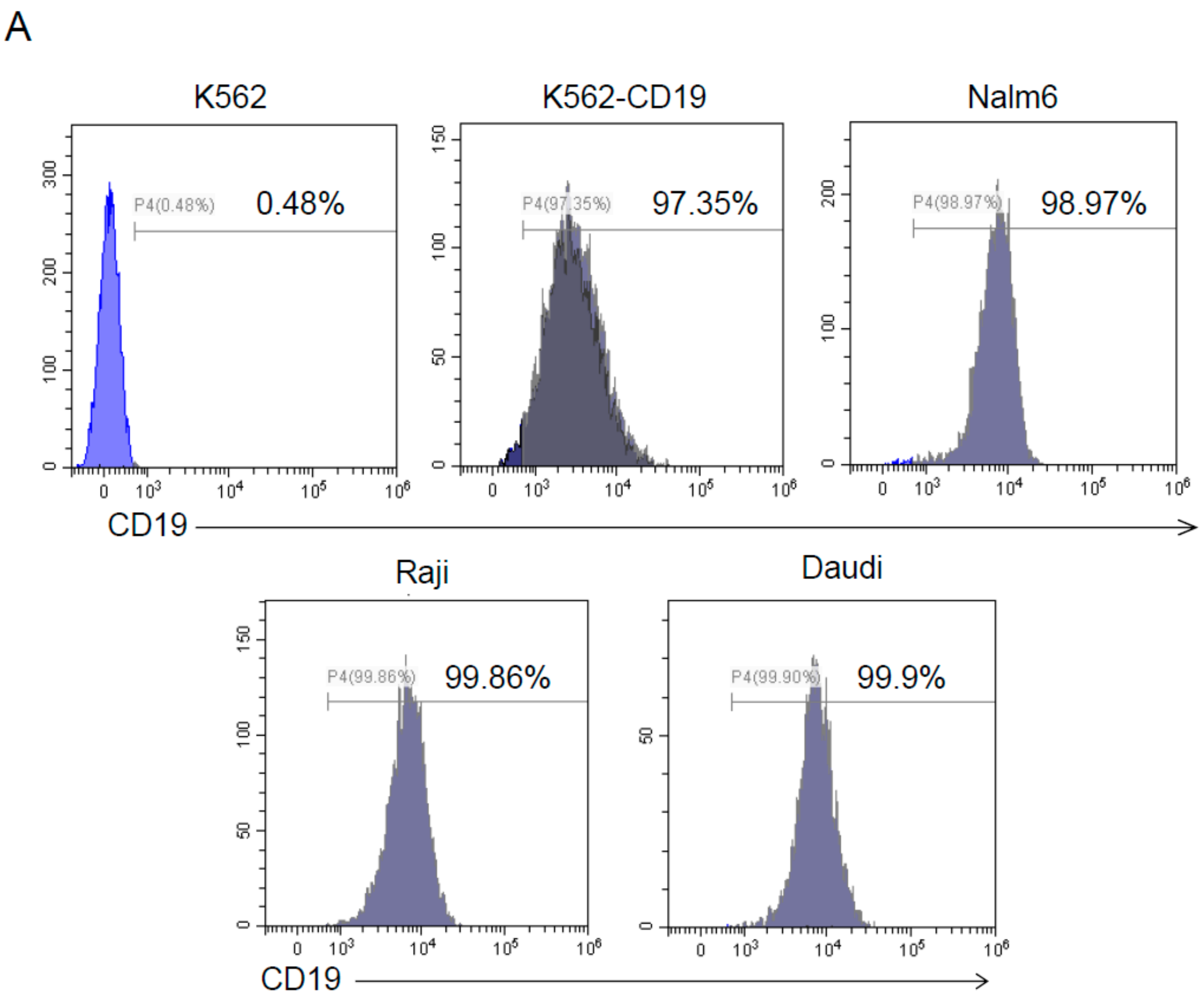

Figure S3. Multiple cancer cell lines expressing CD19 were detected. (A) Flow cytometry analysis showed the CD19 positive rate of different cancer cell lines based on the staining of CD19 antibody. K562 was a negtive control.

Original Photo

Fig 1E Original photo

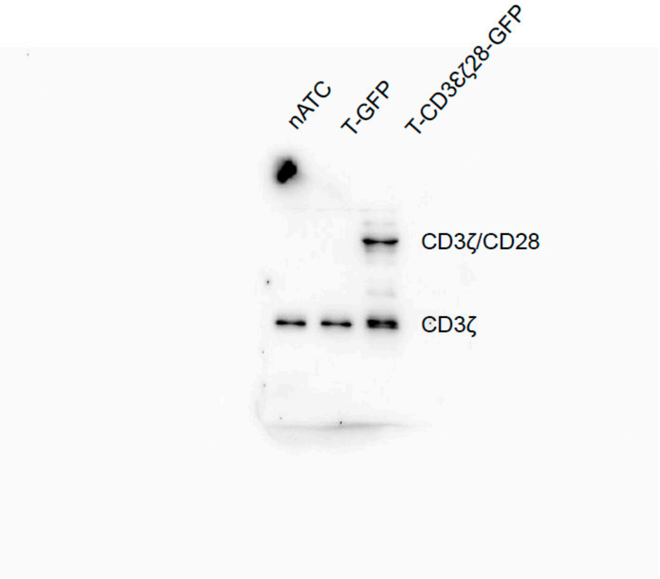

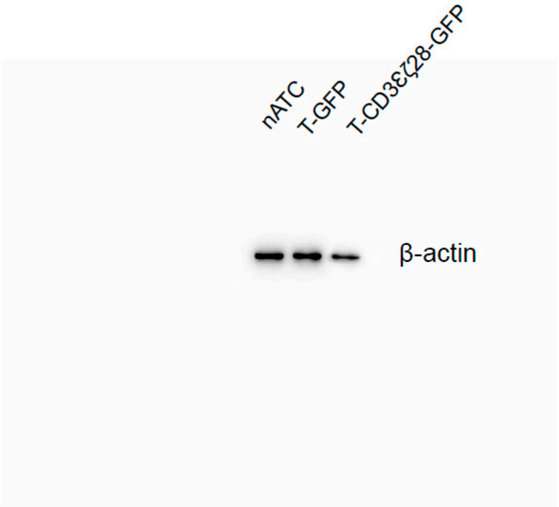

Fig 4C Original photo

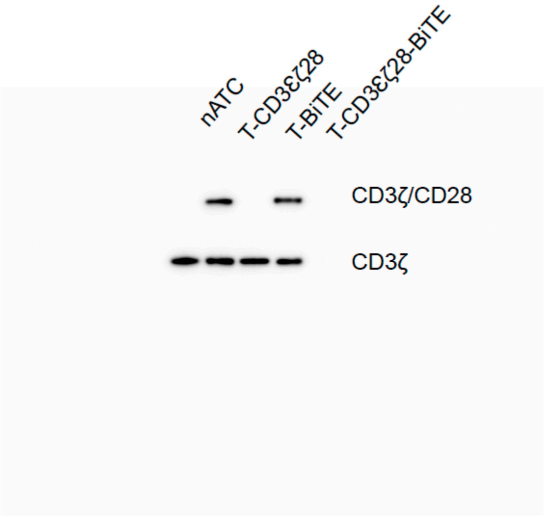

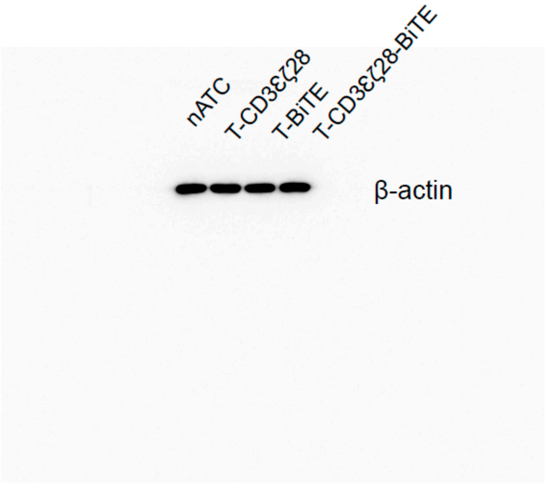

Supplement: Supplementary file 1 [file cancers-14-04947-s001.zip › cancers-1908683-supplementary.pdf]
